# Can surgery relieve pain and act as first-line treatment for a large metastasis of the sternum?

**DOI:** 10.1016/j.ijscr.2019.09.022

**Published:** 2019-09-24

**Authors:** Beatrice Manfredini, Uliano Morandi, Giorgio De Santis, Fabio Catani, Alessandro Stefani, Massimo Pinelli, Alessio Baccarani, Marta Starnoni, Fabrizio Artioli, Beatrice Aramini

**Affiliations:** a Division of Thoracic Surgery, Department of Medical and Surgical Sciences for Children & Adults, University of Modena and Reggio Emilia, Modena, Italy; b Division of Plastic Surgery, Department of Medical and Surgical Sciences for Children & Adults, University of Modena and Reggio Emilia, Modena, Italy; c Orthopaedics and Traumatology Department, University of Modena and Reggio Emilia, Modena, Italy; d Division of Medical Oncology, Ramazzini Hospital, Carpi, Modena, Italy

**Keywords:** Clear-cell renal carcinoma metastasis, Sternal metastasis, Gore-tex mesh

## Abstract

**Background:**

There are few papers published on sternal metastasis from renal cell carcinoma. The unifying element is the operability of the sternal metastasis if it is the only site of metastasis, on the operability of the primary site of the tumor and on the patient’s health conditions.

**Presentation of the case:**

We present a case of a 66-years-old man undergone sternal resection for a large painful metastasis. He was previously undergone left nephrectomy for clear cells carcinoma. *En bloc* resection of the sternal manubrium and right clavicle was performed, a Gore-Tex mesh was placed. Histology confirmed metastasis of kidney clear cells carcinoma. Patient was discharged with no complications and no pain. Chest CT at six months follow up was negative for recurrence.

**Discussion and conclusion:**

We highlighted the importance of surgery as possible *first-line* treatment in symptomatic large sternum metastasis. Therefore, prospective studies should be considered to confirm our strategy.

## Highlights

• Renal cell carcinoma (RCC) is the most frequent type of renal tumor in adults.
• RCC is able to metastasize through the blood system and lymphatic system.
• Metastases from RCC to the bones are often of the osteolytic type.
• The sternal metastatic site for RCC is a rare site and it often begins as pain.

## 1 Introduction

Renal cell carcinoma (RCC) is the most frequent type of renal tumor in adults and is derived from the epithelium of the renal tubules [[1], [2], [3]]. The sternum metastasis is a relatively rare site and it often manifests as swelling of the sternal region, pain or a sensation of tension [4,5]. There are few papers published on sternal metastasis of renal cell carcinoma [[1], [2], [3], [4], [5], [6], [7], [8]]. Aim of our report is to describe a case of a large and painful tumor mass infiltrating the sternal manubrium, undergone radiotherapy for reducing pain with no resolution. He was then undergone surgery. No recurrence after 6 months after surgery was noted. We highlighted the possibility to consider surgery as possible first line treatment in patients with symptomatic metastatic cancer of the sternum.

## 2 Clinical case

**Fig. 1:**
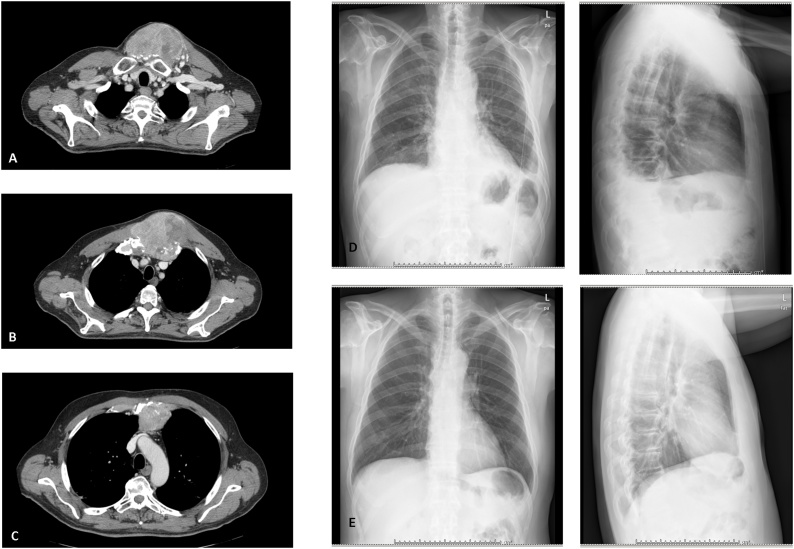
**Radiological assessment before and after surgery.** A-B-C. Chest CT of the sternal metastasis. Fig. 1B shows a clear infiltration of the manubrium. Fig. 1D. Chest x-ray after 2 days from surgery. Fig. 1E. Chest x-ray after 6 months from surgery.

**Fig. 2:**
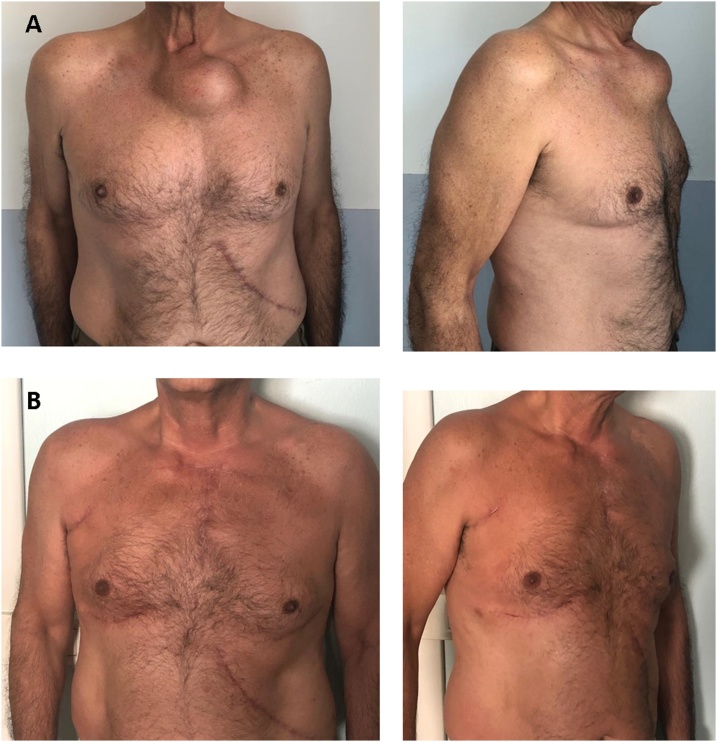
**Clinical evaluation before and after surgery.** A. Frontal and lateral view of the sternal mass before surgery. B. Frontal and lateral view after 6 months from surgery.

A 66-year-old male patient was examined in July 2018 due to painful swelling with increased consistency in the left paramedian sternum at the level of the manubrium. In November 2018 a chest-abdominal computed tomography documented an infiltrating neoformation of the manubrium of the sternum of 54 × 40 mm (Fig. 1A–C), and an 8 mm neoformation in the left kidney. A biopsy of the kidney confirmed the malignancy of the mass, and a left nephrectomy was performed. In December 2018, due to the increasing of size of the sternal mass (Fig. 2A and B) and more pain, an echo-guided needle biopsy confirmed the metastasis of clear cell renal carcinoma. Firstly, the patient underwent 30 Gy transcutaneous radiotherapy. After radiation, ^18^F FGD PET-CT showed a hypermetabolic positivity only at the level of the sternal mass and an increased size (108 × 80 × 90 mm). Due to persistent pain, a multidisciplinary team recommended surgery to remove the sterno-costo-clavicular metastasis.

## 3 Operation technique

**Fig. 3:**
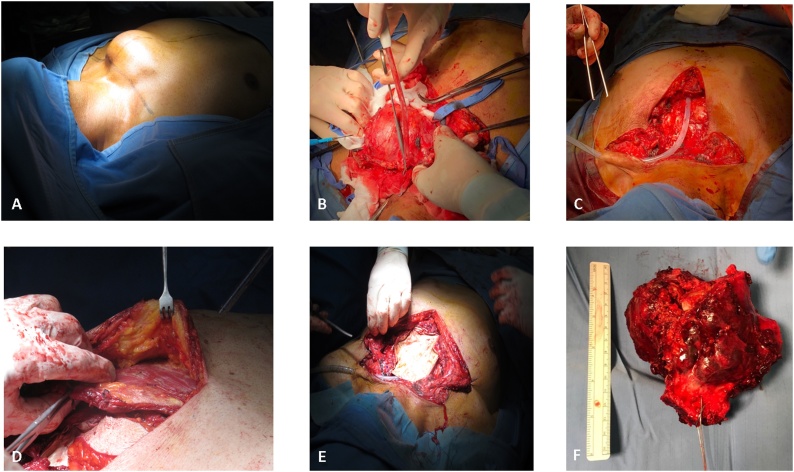
**Steps during the operation.** A. View of the mass before starting the operation. B. Sternal mass removal. C. Drain placement. D. Large pectoralis muscle mobilization before muscle flap transposition. E. Gore-Tex dual mesh placement. F. Sternal mass after surgery.

A cervicotomy and a medial suprasternal longitudinal incision to the distal third of the sternal body were performed. The soft tissue flaps were detached starting with a cervicotomy and progressing to a sternotomy to expose the massive sternocostoclavicular lesion (Fig. 3A and B). Once the sternum was isolated, we proceeded to the cross-section at the body level corresponding to the insertion of the right third rib with a Gigli saw and the same maneuver was performed at the insertion of the second and third left ribs (Fig. 3B). We performed a digital isolation of the sternal insertion of both the clavicle and first rib, protecting the mediastinal vascular plane, proceeding with the initial section of the left first rib and clavicula. A specular maneuver on the right side.

After *en bloc* resection of the lesion (Fig. 3C), a Gore-Tex dual mesh was positioned to cover the underlying structures and fixed with non-absorbable sutures. A right pectoralis muscle flap was harvested including a skin island located at its distal portion (Fig. 3D and E). The humeral insertion of the pectoralis muscle was devided to allow for rotation-transposition of the composite flap to the defect. A left pectoralis muscle flap was also harvested with a similar approach, but without including a skin component. With bilateral mobilization of pectoralis flap a vascularized and thick soft-tissue layer was obtained to fully protect the mesh. The skin was then sutured without tension. A chest x-ray was performed immediately after surgery and after two days from surgery (Fig. 1D).

The postoperative course was characterized by meta-hemorrhagic anemia with 4 units of concentrated red blood cells transfused. The patient was discharged on the nineteenth postoperative day. The histology was positive for clear-cell renal carcinoma with full positivity for PAX8 and mild positivity for TFE3. At six months from surgery, the patient was in good condition with no signs of disease recurrence (Figs. 1E and 2B).

## 4 Discussion and conclusion

RCC is a type of tumor poorly responsive to radiotherapy and chemotherapy treatment; moreover, bone metastases from RCC are often richly vascularized and destructive.

Surgical treatment of these lesions, when the primary tumor can be removed and the operation is allowed by the patient's general condition, can be considered as a first-line treatment because this is the only procedure that can improve the quality of life in terms of survival time and pain relief for these patients. Radiation therapy can be used to reduce the pain caused by metastasis bone infiltration, however, the results on these symptoms are not guarantee, as in our case.

Reconstruction of the thoracic wall with Gore-Tex or polypropylene mesh or with methyl acrylate is currently the most often used and most effective method to ensure effective respiratory mechanics and adequate protection of the underlying mediastinal structures [9].

Most of the published studies describe the possibility of a radical treatment in selected patients who are generally in good conditions, with the only sternal metastasis [[2], [3], [4], [5], [6]]. Although we showed a large mass of the sternum treated with surgery because of the failure of radiation treatment, no recurrence was noted after 6 months. We are conscious of the fact that the follow up period is quite limited; however, we believe that it represents a good proposal for future prospective studies in a larger cohort of patients. This will be necessary to allow this procedure to be used as first-line treatment.

## Sources of funding

No funding.

## Ethical approval

For single case report NO ethical approval needs. Patient signed a consent for publishing the case report.

## Consent

Patient signed a consent for the publication of this case report.

## Author’s contribution

BM and BA wrote the case report. The other Authors read and revised the case report.

## Registration of research studies

Ethical Board approval is not required for case reports in our Center.

## Guarantor

Prof. Uliano Morandi is the Guarantor of this case report.

## Availability of supporting data

Yes.

## Provenance and peer review

Not commissioned, externally peer-reviewed.

## Declaration of Competing Interest

The Authors have no financial and personal relationships to disclose.

## References

[1] Chen S.-C., Kuo P.-L. (2016). Bone metastasis from renal cell carcinoma. Int. J. Mol. Sci..

[2] Cerskute M., Kinčius M., Januškevičius T. (2018). Sternal resection of a solitary renal cell carcinoma metastasis: a case report and a literature review. Acta Med. Litu..

[3] Batista R.R., Marchiori E., Takayassu T.C. (2009). Sternal metastasis as an initial presentation of renal cell carcinoma: a case report. Cases J..

[4] Umer M. (2018). Skeletal metastasis in renal cell carcinoma: a review. Ann. Med. Surg..

[5] Agha R.A., Borrelli M.R., Farwana R., Koshy K., Fowler A., Orgill D.P., For the SCARE Group (2018). The SCARE 2018 statement: updating consensus Surgical CAse REport (SCARE) guidelines. Int. J. Surg..

[6] Pyle Jeremy W. (2005). Sternal resection and reconstruction after renal cell carcinoma metastatic to the sternum. J. Thorac. Cardiovasc. Surg..

[7] Lipinska J. (2017). Chest reconstruction using a custom-designed polyethylene 3D implant after resection of the sternal manubrium. Onco. Ther..

[8] Lee Seock Yeol, Lee S.J. (2011). Sternum resection and reconstruction for metastatic renal cell cancer. Int. J. Surg. Case Rep..

[9] Harati K., Kolbenschlag J., Behr B., Goertz O. (2015). Thoracic wall reconstruction after tumor resection. Front. Oncol..

